# HLA-G: An Important Mediator of Maternal-Fetal Immune-Tolerance

**DOI:** 10.3389/fimmu.2021.744324

**Published:** 2021-10-29

**Authors:** Baimei Zhuang, Jin Shang, Yuanqing Yao

**Affiliations:** ^1^Medical School of Chinese People’s Liberation Army, Chinese People’s Liberation Army General Hospital, Beijing, China; ^2^Shenzhen Key Laboratory of Fertility Regulation, The University of Hong Kong-Shenzhen Hospital, Shenzhen, China; ^3^Department of Obstetrics and Gynecology, The First Medical Centre, Chinese People’s Liberation Army General Hospital, Beijing, China

**Keywords:** HLA-G, maternal-fetal immune-tolerance, reproductive immunology, KIR receptor, pregnancy

## Abstract

Maternal-fetal immune-tolerance occurs throughout the whole gestational trimester, thus a mother can accept a genetically distinct fetus without immunological aggressive behavior. HLA-G, one of the non-classical HLA class I molecules, is restricted-expression at extravillous trophoblast. It can concordantly interact with various kinds of receptors mounted on maternally immune cells residing in the uterus (e.g. CD4+ T cells, CD8+ T cells, natural killer cells, macrophages, and dendritic cells) for maintaining immune homeostasis of the maternal-fetus interface. HLA-G is widely regarded as the pivotal protective factor for successful pregnancies. In the past 20 years, researches associated with HLA-G have been continually published. Indeed, HLA-G plays a mysterious role in the mechanism of maternal-fetal immune-tolerance. It can also be ectopically expressed on tumor cells, infected sites and other pathologic microenvironments to confer a significant local tolerance. Understanding the characteristics of HLA-G in immunologic tolerance is not only beneficial for pathological pregnancy, but also helpful to the therapy of other immune-related diseases, such as organ transplant rejection, tumor migration, and autoimmune disease. In this review, we describe the biological properties of HLA-G, then summarize our understanding of the mechanisms of fetomaternal immunologic tolerance and the difference from transplant tolerance. Furthermore, we will discuss how HLA-G contributes to the tolerogenic microenvironment during pregnancy. Finally, we hope to find some new aspects of HLA-G in fundamental research or clinical application for the future.

## 1 Introduction

Three decades ago, a unique human leukocyte antigen (HLA) like molecule, named HLA-G, was found to be highly expressed in extravillous trophoblast (EVT). Today, HLA-G is now established as a key molecule that plays an important role in the generation of fetal-induced maternal immunological tolerance (1, 2). Through Southern blot hybridization analysis in 1982, HLA-G was first detected as an uncommon HLA class I gene on human chromosome 6 (3). Soon after, the gene encoding HLA-G was isolated from a genomic DNA library constructed from the B-LCL 721.11 cell line and initially named HLA-6.0 (4, 5). DNA sequencing revealed that HLA-G had a similar exon/intron structure and an 86% overall homology at the protein level with the classical class I genes HLA-A, -B, and –C, but a 5’ flanking region with distinctive 5’ regulatory elements. In 1994, functional studies showed that HLA-G inhibited decidual natural killer (dNK) cell killing and two years later, HLA-G was also found to inhibit peripheral natural killer (pNK) cell cytotoxic activity (6, 7). In 1999, the killer immunoglobulin-like receptor 2DL4(KIR2DL4) was described as a ‘universal’ HLA-G receptor on NK cells for all KIR haplotypes (8). These initial studies established the foundation of subsequent research on the role of HLA-G in establishing maternal-fetal immune-tolerance during early pregnancy.

### 1.1 Structure, Alternative Splicing Variants, and Translated Isoforms of HLA-G

HLA-G is a limited polymorphic major histocompatibility complex (MHC) molecule, with only 88 known alleles and 26 protein variants recorded in the IMGT/HLA sequence database (http://www.ebi.ac.uk/imgt/hla/stats.html, September 2021). The gene structure of HLA-G is highly similar to the other HLA class I genes with eight exons and seven introns and encodes a hetero-trimer protein isoform (3). Exon 1 encodes signal peptides whereas exons 2, 3, and 4 encode the extracellular domains α1, α2, and α3, respectively. Exons 5 and 6 encode the transmembrane and cytoplasmic domains, however, a stop codon in exon 6 impedes the formation of the mature mRNA encoded by exon 7, and thus exon 8 is not translated (9).

Different from classical MHC class I genes, the HLA-G gene promoter region harbors some conserved but defective regulatory motifs consisting of enhancer A, interferon-stimulated response element (ISRE), and the SXY module (S: unknown factor, X1: regulatory factor X, X2: cAMP response element-binding protein, Y: nuclear factors Y). Its enhancer A does not bind the p65 subunit of NF-kappa B to respond to tumor necrosis factor –α(TNF-α) signaling and the ISRE is unresponsive to IFN-γthrough bonding to IRF1. Likewise, the SXY module contains S, X1, and defunct X2 and Y elements. These non-functional elements are important for the constitutive and CIITA-mediated transactivation of MHC class I genes *via* recognition by ATF1/CREB1 transcription factors and the RFX complex (10, 11). Nevertheless, neither NLRC5 nor CIITA are expressed in EVT. Recently, Enhancer L, a cis-regulatory element 12 kb upstream of HLA-G with enhancer activity, was discovered using a massively parallel reporter assay (MPRA) (12). Ferreira LM et al. discovered that a long-range chromatin looping interaction between Enhancer L and the HLA-G classical promoter, controlling the placental-specific HLA-G expression. The interaction is mediated by core trophoblast transcription factors of the CEBP and GATA families. The long interspersed element-1 (LINE1), located 4 kb upstream of the HLA-G classical promoter named gL, provides an AT- or A-rich region to form hairpin loops that repress HLA-G expression in non-trophoblast cells (13). Other identified alternative regulatory elements including heat shock, progesterone, and hypoxia-responsive elements also regulate the transcription of HLA-G. The unidentified responsive elements for IL-10 and glucocorticoids are under-identified in the HLA-G promoter. There are also a variety of single nucleotide polymorphism (SNPs) in 3’ untranslated region (UTR) of HLA-G that influence mRNA stability, turnover and mobility, including +3035C/T, +3187A/G, +3196C/G, +3142C/G, +3001C/T, +3003C/T, +3010C/G, +3027C/A, +3032C/G, +3052C/T, +3092G/T, +3111A/G, +3121C/T, +3177G/T, +3183A/G, and +3227A/G (14, 15). These SNPs alter the binding of microRNA (miR), leading to decreased expression of HLA-G. Six types of miRs have been identified, namely; miR148a, miR148b, miR152, miR133a, miR628-5p, and miR548q. Nevertheless, none of these miRs specifically target HLA-G since they can also bind to 3’UTR of other HLA class I mRNAs. Interesting, miR152 has been shown to repress trophoblast-specific HLA-G expression without affecting trophoblast-important invasion ability and the up-regulation of NK cell-mediated cytolysis of host cells (16). Moreover, 14bp insertion/deletion, an indel variant between positions +2961 and +2974 in the 3’UTR, modulates HLA-G expression and splicing patterns (17). In addition, epigenetic modifications such as DNA or RNA methylation and histone deacetylation can impact the expression of the HLA-G gene (18, 19). Therefore, the transcriptional and post-transcriptional regulation of HLA-G is under tight tissue-specific regulation.

**Figure 1:**
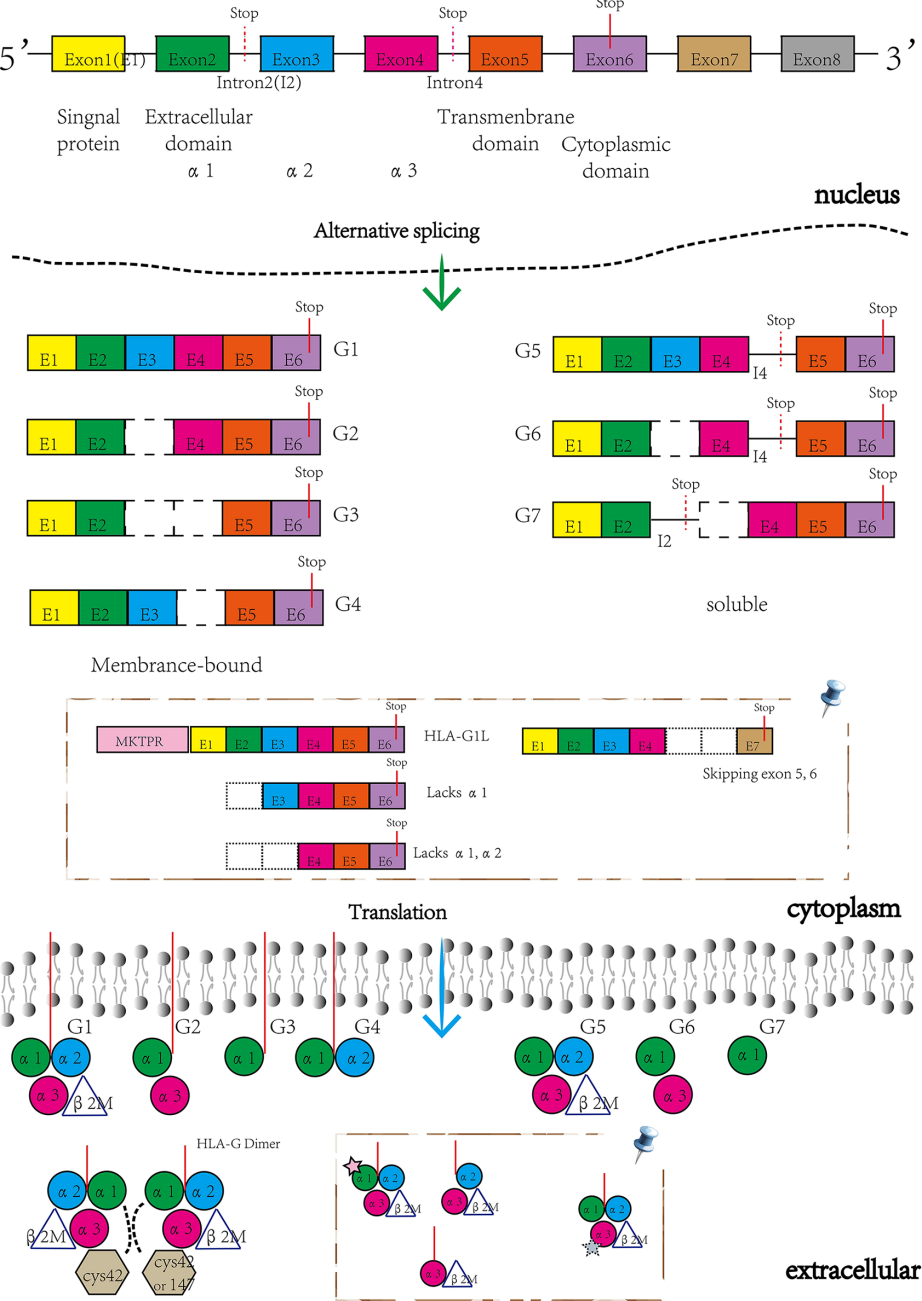
The structure of HLA-G gene and its encoding isoforms generated by alternative splicing mRNAs. Novel HLA-G isoforms reported by Tronik-Le Roux D et al. (20) (pin). Membrane-bound isoforms included HLA-G1L with a 5’-extended end of five additional amino acids (MKTPR), the lacking α1 domains and the only α3 domains, while the soluble isoforms skipping exon 5 and 6.

**Figure 2:**
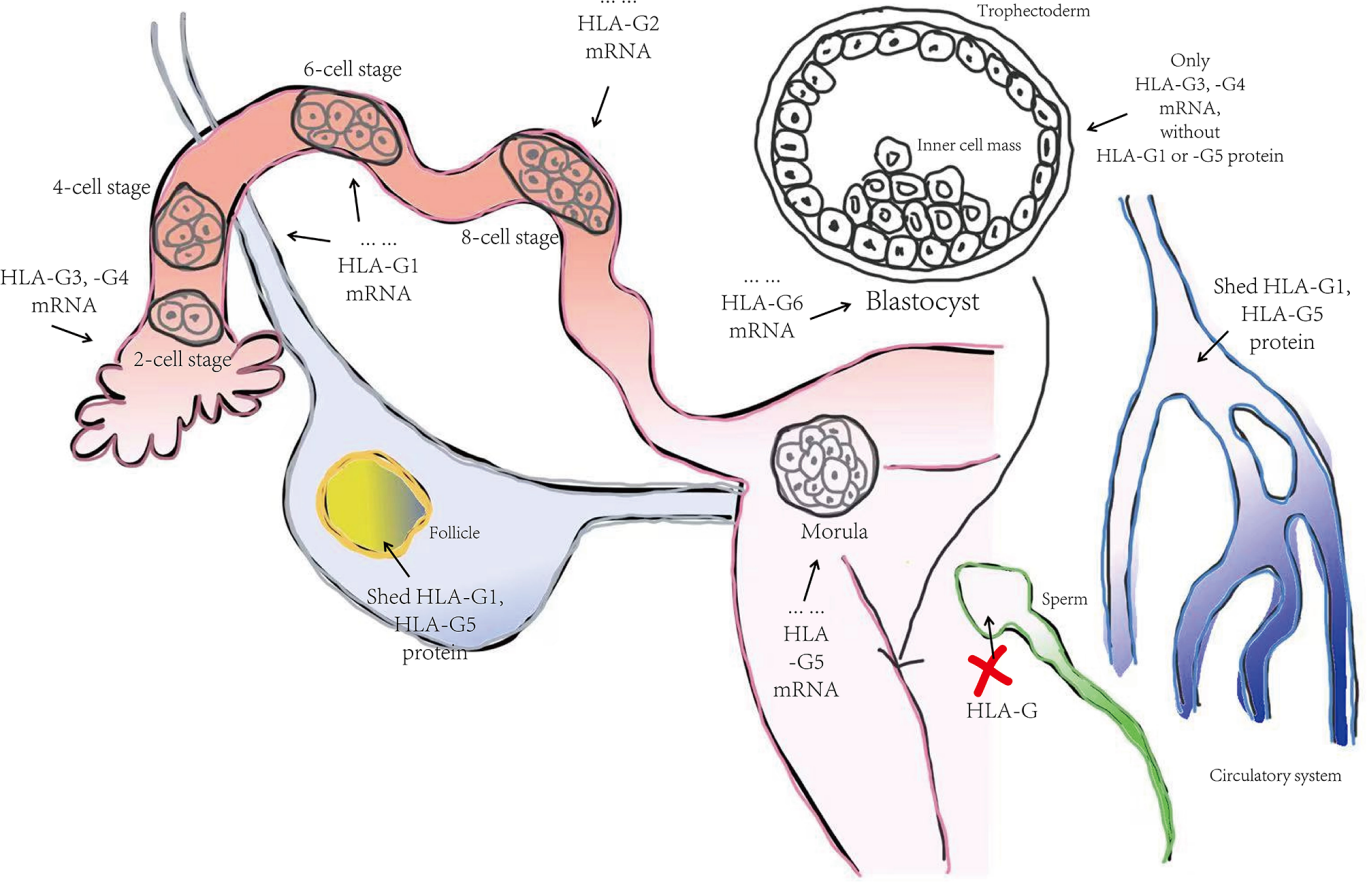
The progression of HLA-G molecule expression in the human preimplantation stage. Summarized from Yao YQ et al. (2005) (22). HLA-G3 and –G4 represent the predominant spliced transcripts in the whole preimplantation stage, followed by HLA-G1, -G2, and -G5. HLA-G6 did not appear until the blastocyst stage. After implantation, HLA-G1 and –G5 were expressed toward differentiation into trophectoderm and gradually faded away in the inner cell mass during development. Interestingly, shed HLA-G1 and HLA-G5 were detected in follicle and female blood but not in male gametogenic cell-like sperm.

Despite complex regulation of the HLA-G gene, transcription only yields seven alternative mRNAs that encode seven different protein isoforms, namely; membrane-bound (HLA-G1, -G2, -G3, -G4) and soluble (HLA- G5, -G6, -G7) molecules (see **Figure 1**). Only the HLA-G1 and -G5 mRNAs encode the full-length HLA-G isoform associated with β2-microglobulin (β2M), with the HLA-G5 mRNA retaining intron 4, while the others lack globular domains for binding β2M. Moreover, the membrane-bound HLA-G1 can produce a soluble form named shed HLA-G1, mediated by metalloproteinase cleavage (21). Introns 2 and 4 can prevent translation of the transmembrane domains and the cytoplasmic tails due to the presence of a premature stop codon. Thus, HLA-G5, -G6 and -G7 mRNA encode soluble types. The heterozygotes for the HLA-G allele had been found to relate to levels of these transcripts. G*01012 allele decreases the level of HLA-G1, -G2, and –G3. G*01013 allele increase the levels of HLA-G2 and -G4. Human preimplantation embryos express some of the different alternative HLA-G mRNAs when evolving (22), although the truncated HLA-G3 and G4 mRNAs represent the predominant spliced transcripts (see **Figure 2**). HLA-G5 was not expressed until the morula stage and was poorly expressed versus HLA-G1 which was majorly expressed in blastocysts. The presence of HLA-G mRNA has related to a higher cleavage rate at the blastocysts stage (23). HLA-G1 and –G5 protein was majorly expressed in the hatching blastocyst which was in contact with the endometrium at implantation. After implantation, HLA-G1 and –G5 were expressed toward differentiation into trophectoderm (TE). They also had been detected in early inner cell mass (ICM) but down-regulated during development. Surprisingly, the HLA-G1, -G2, and –G6 isoforms are preferentially expressed only in invading cytotrophoblasts, such as the terminal edge of trophoblast columns and the chorion membrane cytotrophoblasts cells, rather than syncytiotrophoblast or other villous cytotrophoblasts. However, whether the expression of HLA-G2 and –G6 is presented in endovascular EVT still remains controversial. HLA-G5 was ubiquitous in trophoblast subpopulations (24, 25). HLA-G4 and HLA-G7 putative protein products remain unknown due to their mRNAs being scarcely detected in placentas. Meanwhile, HLA-G3 has no specifically identified antibodies, and should be present in placentas. Hence, although soluble HLA-G (sHLA-G) is produced by some but not all preimplantation embryos, sHLA-G secretion is still a prerequisite for successful implantation recently, and thus shed HLA-G1 and –G5 levels can potentially influence pregnancy outcomes (26, 27).

Under normal physiological conditions, HLA-G has a restricted expression in immunologically privileged sites, such as EVT, cornea, thymus, proximal nail matrix, erythroblasts, and mesenchymal stem cells. The functional protein is a hetero-trimer formed by β2M, an alpha heavy chain, and a peptide antigen. The heavy chain consists of three extracellular globular domains α1, α2, and α3, a transmembrane region, and a short cytoplasmic domain. Interestingly, while the heavy chain is anchored in the membrane and approximately 45kD, detected HLA-G isoforms are 39kD in size. Furthermore, the heavy chain has two single cysteine residues at positions 42 (Cys42-Cys42 bonds) and 147 (Cys42-Cys147 bonds). These sites can interact with each other to generate disulfide bonds and thus form the HLA-G dimer (see **Figure 1**). Several studies have shown that HLA-G dimers, compared to monomers, can bind to their receptors (especially, immunoglobulin-like transcript (ILT) receptor 2) with higher affinity and exhibit slower dissociation rates (28, 29). Notably, Tronik-Le Roux D et al. predicted some novel HLA-G isoforms from a transcriptome analysis in renal cancer lesions (20) (see **Figure 1**). For further exploration into which bio-functional contexts the HLA-G protein is performed across different pregnancy stages concerning the maternal-fetal immune phenotype, commercially available HLA-G antibodies for different alternative isoforms should be developed and produced. Also, higher quality with less cross-reaction is needed, since MEM-G/1 and 4H84 reportedly have cross-reactivity with other HLA class I molecules. **Table 1** shows the HLA-G recognizing antibodies and their specificity and applications currently. The expression of HLA-G protein is also under strict regulation. There are a lot of significant regulated factors including interferons, interleukin (IL)-2, IL-10, granulocyte macrophage-colony stimulating factor (GM-CSF), tumor necrosis factor β (TGF-β), hypoxia, and indoleamine-2,3-dioxygenase (IDO). These micro-environmental factors were present in maternal-fetal interplay to affect the HLA-G expression and sequentially its biological function.

**Table 1:** HLA-G antibodies and their specificity and applications. Updated from Krijgsman D et al. (2020) (30) and Lin A et al. (2018) (31).

HLA-G mAbs	Immunogen	Specificity	Applications
MEM-G/1	Denatured bacterially expressed recombinant human HLA-G heavy chain	Denatured HLA-G heavy chain, all isoforms	IHC(F/P), WB
MEM-G/2		Free heavy chain of all HLA-G isoforms	
MEM-G/9 (IgG1)	Recombinant full length protein corresponding to human HLA-G	Native form of HLA-G1 and HLA-G5 isoform associated with β2M, Reactivity with HLA-G3 was reported	IHC(F), IP, FC, ELISA
MEM-G/11		HLA-G1	IHC(F), ICC, IP, FC, ELISA
01G	HLA-B27 transgenic mice immunized with H-2 identical murine cells transfected with HLA-G and human β2-microglobulin	Full-length HLA-G1	
87G (IgG2a)		HLA-G1 and HLA-G5, blocks interaction of HLA-G with inhibitory receptors	IHC(F), FC, ELISA, FUNC
2A12	C-terminal amino acid sequence (22-mer) of soluble HLA-G5 and HLA-G6 proteins coupled to ovalbumin	HLA-G5 and HLA-G6	IHC(F/P),FC, WB, ELISA
5A6G7 (IgG1)			IHC(F/P), ICC, FC, WB, ELISA
4H84 (IgG1)	Amino acids 61-83 of HLA-G α1 domain of human origin	An epitope in the HLA-G α1 domain	IHC(P), ICC, IP, WB, ELISA
G233 (IgG2a)	Murine L cells transfected with both human beta2-microglobulin and HLA-G	Several isoforms of HLA-G expressed in all populations of extravillous trophoblast. Without cross-react with any other MHC Class I antigens (HLA-A, -B, -C, -E, -F)	ICC/IF
MEM-G/4	Recombinant human HLA-G denaturated heavy chain	denaturated HLA-G heavy chain	WB
6D463			IHC(F/P), IP, WB
9-1F10	Recombinant soluble HLA-G of human origin	HLA-G5 and HLA-G6	IF, IP, WB, FC

### 1.2 Bio-Functional Character of HLA-G

Tolerogenicity is the major biological function of HLA-G during fetal development. HLA-G expressed by invading EVT serves as a potential ligand for directly binding to cell surface receptors of leukocytes, including NK cells, T cells, B cells, and antigen-presenting cells (APC) residing in the maternal decidua. These inhibitory or activating receptors include ILT-2 (CD85j; LILRB1), ILT-4 (CD85d; LILRB2), CD8, and KIR2DL4 (CD158d) (32–35) (see **Table 2**). Besides this, CD94/NKG2A previously was considered an inhibitory receptor of HLA-G, but more recently it has been shown to target HLA-E molecules in situations where the HLA-G signal peptides were presenting to HLA-E molecules (36, 37). A further study has also shown that the ligand-receptor assignment between HLA-G and CD94/NKG2A is dependent on the amino acid composition of the HLA-G heavy chain (38).

**Table 2:** Overview of receptors for HLA-G and their interactional role.

Receptors	Immune cell type	Interactional role
IL2	T cell	Inhibition of proliferation, cytolysis, chemotaxis and cytotoxicity; Inhibition of IFN-γ secretion of γδT cells Induction of Tregs and Th2-type cytokine; Induction of IL-10, IL-4 and TGF-β secretion
	B cell	Inhibition of proliferation, chemotaxis and Ig secretion
	NK cell	Inhibition of cytotoxicity and chemotaxis; Inhibition of MICA/NKG2D activation; Inhibition of IFN-γ secretion
	dendritic cell	Inhibition of maturation; Induction of tolerogenic DC and anergy and suppressor T cell; Induction of IL-10 and TGF-β secretion
	IL-10-differentiated DC	Induction of Tregs
	myeloid-derived suppressor cell	Induction of Tregs
	macrophage	M2 bias; Induction of IL-10, IL-4 and TGF-β secretion
IL4	dendritic cell	Inhibition of maturation; Induction of tolerogenic DC and anergy and suppressor T cell
	IL-10-differentiated DC	Induction of Tregs
	myeloid-derived suppressor cell	Induction of Tregs
	macrophage	M2 bias; Deviation of APC to tolerogenic phenotype
	Neutrophils	Inhibition of reactive oxygen species production and phagocytosis
KIR2DL4	NK cell	Induction of transcytosis, apoptosis and active Akt pathway; Induction of protective factors secretion; Induction of TNF-α, INF-γ, IL-10 and IL-1β secretion; Inhibition of killing targeted cell
CD8	CD8+T cell	Inhibition of proliferation, chemotaxis and cytotoxicity; Inhibition of IFN-γ secretion
CD160	Endothelial cell	Angionesis

Some co-stimulatory molecules such as programmed cell death protein-1 (PD-1), cytotoxic T-lymphocyte-associated protein (CTLA-4), T-cell immunoglobulin, mucin-domain containing-3 (TIM-3), and factor associated suicide (Fas; CD95) are intimately involved in tumor-driven immune escape mechanisms, due to their moderating effect on the activation, proliferation, and differentiation of T cells. HLA-G has a synergetic relationship with them and is thought to be a new immune checkpoint (IC) molecule. In a study on endometriosis-associated infertility, Santoso B et al. have investigated the level of soluble(s) CTLA, sPD-1, sPD-L1, and HLA-G in endometriosis. HLA-G may act similar to IC molecules presented on cytotoxic T cells (CD8+T cells), they have found HLA-G accompanied sPD-L1 and sCTLA-4 may implicated in the pathological mechanisms of endometriosis-related infertility (39). Schwich E et al. demonstrated that sHLA-G1 significantly increased the frequency of ILT-2 receptors on CD8+ T cells and might be involved with other IC molecules to generate an immunosuppressive or exhausted phenotype (40).

ITL-2 is also anchored on dNK cells and thus can be potentially regulated by sHLA-G1. Studies reveal that the β2m-linked isoforms or disulfide-linked dimers of HLA-G execute the immune-associated function whereas free β2m has no immune function (35, 41). Gonen-Gross et al. clarified that HLA-G free H chain complexes, which form without β2m, are present on the trophoblast cell surface but unimportant for ILT2 recognition (28). However, these HLA-G complexes modulate the cytotoxicity of NK cells *via* interaction with ILT2 (28). ILT4 is a predominantly immunosuppressive molecule presenting on dendritic cells (DCs), one of the major APCs. Cai Z et al. demonstrated that ILT4 interference modulated HLA−G expression in a dose−dependent manner (42). Furthermore, ILT4 interacted with HLA-G to regulate cell proliferation, invasion, and migration of CRC by activating protein kinase B (Akt) and extracellular signal−regulated kinase (Erk) signaling (42).

DC-10, a subpopulation of DCs which secrete high levels of IL-10, can induce Type I T regulatory cells depending on ILT4/HLA-G signaling pathway (43). Myeloid-derived suppressor cells (MDSCs) could stimulate *via* sHLA-G through its autonomous expression of ILT2 and ILT4. These activating MDSCs subsequently undergo phosphorylation of the signal transducer and activator of transcription 3 (STAT3), as well as induction of IDO. KIR2DL4, one of the members of the killer immunoglobulin-like receptor (KIR) family, is expressed on decidual NK cells. Different from other KIR members, KIR2DL4 is basically expressed in all NK cells and has dual-function either inhibiting or activating regulation. Studies have shown that sHLA-G interacts with KIR2DL4 expressed by primary or resting NK cells in an endocytosis manner (44, 45). This in turn actives the signaling pathway associated with a pro-inflammatory and pro-angiogenic response (DNA-PKcs, Akt, NF-kappa B) and induces NK cell apoptosis or functional inability. However, HLA-G as the ligand for KIR2DL4 during pregnancy has been studied under the circumstance of trophoblasts-dNK cells interactions (46). As for the inhibitor for the killing activity of NK cells encountering HLA-G positive fetuses, the decrease of KIR2DL4 expression seems to be a risk for the increased susceptibility of NK cell-mediated cytotoxicity and subsequent recurrent spontaneous abortion. Thus, HLA-G interacts with various kinds of immune cells through their receptors to induce a secretory or suppressive phenotype and thus acts primarily as a molecular switch in immunological tolerance.

Anti-HLA-G antibodies are found only in women who have achieved at least one pregnancy experience (47). Interestingly, HLA-G only acts as a physiological antigen during pregnancy. The biological function of trophoblast-specific HLA-G may not be confined to the tolerogenic properties. Other biological characteristics of trophoblast HLA-G have also been considered, including secretion of chemokines or hormones, angiogenesis for the nourishing fetus, adhesion, and invasion related to endometrial receptivity (48). Incidentally, *via* using a rabbit corneal neovascularization model, BY55/CD160, which is a glycosylphosphatidylinositol-anchored receptor expressed by endothelial cells, was found to be a new soluble HLA-G receptor for the inhibition of FGF2-induced angiogenesis (49). Thus, it may provide more significant functional details of the HLA-G gene when excavating the biological features beyond immunity, such as angiogenesis.

Previous studies by our team have identified some biological properties of HLA-G beyond immune tolerance. For example, HLA-G promotes trophoblast fusion and β-hCG production through the Erk1/2 pathway in JEG-3 cells (50). HLA-G5 isoforms stimulate invasion of primary trophoblasts and JEG-3, JAR cells by activating phosphorylation in the ERK pathway (51). In addition, *in vitro* studies showed that HLA-G1 inhibits JAR and HTR8/SVneo cell invasion induced by hepatocyte growth factor (HGF) and correlates with the oxygen conditions in the cultivated microenvironment (52).

Understanding how HLA-G influences maternal-fetal immune-tolerance is still a significant subject of research. Hence, this review aims to summarize the biological features of HLA-G and our understanding of the mechanisms of fetomaternal immunologic tolerance. We also discuss the differences with transplant tolerance. Furthermore, we will discuss how HLA-G contributes to the tolerogenic microenvironment during pregnancy. Finally, we also explore HLA-G potential for clinical application in pregnancy.

## 2 Immunological Tolerance and Maternal-Fetal Immune-Tolerance

A genetically distinct fetus resembles a semi-allogeneic antigenically foreign body to the mother. However, it can be maintained and developed in the expectant mother over a long-term gestational period. It seems pregnancy defies the basic tenets of immunology. However, in some pregnancies, the failure of fetomaternal immunologic tolerance is correlated with miscarriage, preeclampsia, and other pathological conditions (53). Therefore, the establishment of successful pregnancy poses an immunological challenge to the balance of maternal-fetal interplay.

### 2.1 The Mechanism of Immunological Tolerance

Generally speaking, immunological tolerance is intricate. How is the unresponsiveness of the adaptive immune system to self-antigens established and maintained? How is the quality and magnitude of adaptive immune responses to non-self-antigens controlled to avoid damage to the host? To our knowledge, the mechanisms of immunological tolerance can be concluded as follows.

T and B cells are the basis of immunological tolerance (54–56). To tolerize self-antigens, T and B cells first experience central tolerance and subsequently form peripheral tolerance. The developing T and B cells undergo negative selection. Specific immune clones are then induced succeeding apoptosis (that is, clonal deletion) and inactivation (that is, clonal anergy) under the stimulation of autoantigen. The self-reactive T and B cells that escape negative selection are exported to the peripheral circulation. Some cells cannot be activated due to a low concentration of antigen, which is referred to as immunological ignorance. Other cells undergo clonal deletion or clonal anergy when repeatedly stimulated by antigen without a co-stimulatory signal. The remaining cells are inhibited by a variety of negative regulatory mechanisms including Treg cells (57, 58). However, this type of autoimmune tolerance can break down for diseases through pathogen-associated molecular patterns (PAMP) or alternative activated pathways of DC and Th cells (59). Certainly, to tolerize non-self-antigens, the main mechanism is a lack of efficient co-stimulation signal and a high level of Treg cells.

Regulatory T (Treg) cells, referred to as CD4+CD25+Foxp3+ T cells, are well-accepted to be one of the key inducers of immunological tolerance (60). They are derived from autoreactive T cells and negatively regulate immune responses through two following patterns: (1) direct connection to suppress targeted cell activation; (2) secrete cytokines (e.g. TGF-β, IL-10) for protection. However, a subpopulation of autoreactive B cells undergo additional modifications called receptor editing, that is, reboot gene rearrangement of immunoglobulin, and thus generate another B cell clone with a new BCR *via* another light-chain gene (61). Regulatory B cells, Regulatory DC cells and myeloid-derived suppressor cells are also valuable for immunological tolerance.

Incidentally, immunologically privileged sites cannot be ignored. Immunological tolerance might arise here on account of: (1) physiological barrier, as well, (2) partial microenvironment, (3) inducing apoptosis of Fas+ lymphocyte by expressed Fas ligand, and (4) suppressing T cell response by generating inhibitory cytokines centered on TGF-β or expressed PD-1 ligands.

Immunological tolerance has high specificity (62). It was non-responsive to specific antigens while still well-responsive to other antigens, which is different from immunosuppression and immunodeficiency. The placenta is a more specific immunologically privileged site that prevents the fetus that contains paternal heredity from maternal immunological attack. It correlates with diversified factors to establish and maintain maternal-fetal immune-tolerance. HLA-G was restrictedly expressed in EVT, which is the functional unit of the placenta, under the physiological status and well-accepted the key mediator of fetomaternal immunologic tolerance.

### 2.2 Medawar’s Paradox and Mother-Baby Immunological tolerance

In brief, immunological tolerance is defined as a state of immune anergy where the immune system fails to respond to specific antigens. Different from immunosuppression or immunodeficiency, fetomaternal immunological tolerance does not affect the integrated ability of adaptive immune response. This poses several fundamental questions. First, how does the maternal immune system recognize the fetus-derived antigens as ‘self-antigen’ rather than ‘non-self-antigen’? Second, how does the fetal immune system adapt to maternal circulatory antigens without compromising its own development? Third, what happens during the immune trafficking between mother and baby? Fourth, what is the process of inducting or forming tolerogenesis at the maternal-fetal interface? Lastly, why is this type of immunological cross-talk highly specific, and why does it only occur locally and appear not to disrupt the maternal immune cycle? The underlying mechanisms are still enigmatic and perplexing.

In 1945, Owen first reported contact with an alloantigen can induce immunological tolerance. It was observed that skin transplant rejection between fraternal twin derivative calves from one to another did not occur resulting from erythrocyte chimera (63). It was proposed that immune cells are immature in their early stage of development and can be artificially induced to tolerate ‘non-self’ antigens. In 1961, MacFarlane Burnet, who put forward the clonal selection theory, believed that the immature autoimmune responsive cells that contact self-antigens develop tolerance *via* clonal sweeping (64). Seemingly, those theories of natural immunological tolerance were well accepted in the field. But, how is acquired immunological tolerance generated in pregnancy? Why are some women who experience miscarriage at a high risk of subsequent miscarriages? How can embryos derived from donated oocytes or embryos for gestational surrogacy survive in a genetically mismatched maternal microenvironment and develop into healthy babies? The famous ‘Medawar’s Paradox’ proposed by Dr. Peter Medawar, who achieved the Nobel Prize for the discovery of immunologic tolerance, has tried to answer the question: why a can mother accept her baby without immunosuppression? This immunological paradox is based on three hypotheses: anatomical separation, immaturity of fetal antigens, and maternal immunological inertness (65).

#### 2.2.1 Anatomical Separation: Placenta-Decidua Barrier

An orchestrated sequence of events dynamically unfolds post-implantation and during pregnancy, involving decidualization of endometrium and a symbiotic development of trophectoderm (TE) (66–69). Decidua is supported by progesterone and only appears in pregnancy. There is an influx of leukocytes, such as decidual NK (dNK) cells, macrophages (Mφ), and Treg cells, recruited at the maternal-fetal interface. They collectively confer tolerance to fetal antigens and help maintain a homeostatic environment conducive to fetal survival and development. These maternally derived immune cells are typically distinct in phenotype and function from their circulating peripheral counterparts.

Highly granulated CD56+CD16- dNK cells account for 70% of the immune cell population at the maternal-fetal interface (70). They secrete a series of cytokines, growth factors, angiogenic factors, and immunomodulatory molecules to enhance decidua receptivity, including type II interferon (IFN-γ), GM-CSF, TGF-β, IL-8, IL-10, IL-13, and CX-chemokine ligand-10 (CX-CL10). Functional studies have shown that dNK cells are not directly cytolytic towards fetal EVT cells (71). The dNK cells express an abundance of receptors and can bind to several ligands selectively expressed in EVT, such as HLA-C, HLA-G, HLA-E, and HLA-F. Intriguingly, dNK cells can be induced to highly express growth factors and immunomodulatory proteins by multi-gravidity (72, 73). Decidual Mφs (dMφs) are another type of APC comprising up to 20%-25% of the CD45+ antigen-presenting population and are phenotypically characterized as CD163+CD206+DC-SIGN+. dMφs have highly versatile functions including regulation of adaptive T cell responses, monitoring innate NK cell responses, vascular remodeling, tissue regeneration, and fetal antigen recognition. They predominantly produce IL-10, prostaglandin E2 (PGE2), and IDO and are proficient in scavenging by phagocytosing apoptotic trophoblasts (74). On this basis, they inhibit the activation of pro-inflammatory pathways. T cells are also present at the maternal-fetal interface and their composition alters during pregnancy. In the decidual microenvironment, CD4+ T cells exhibit an activated/memory CD25dim phenotype, whereas CD8+ T cells exhibit an effector/memory CD28- cell surface phenotype. The balance of the helper T (Th) 1 and Th2 cells changes trending towards an abundance of Th2 cells in the early stage is thought as a maker for successful pregnancy (75). The Treg cell population expresses intracellular fork-head box transcription factor (FOXP)-3 and activates immature DCs which in turn participate in immunomodulatory and proangiogenic functions (76). Likewise, the nonimmune cells, such as endothelial cells, stromal cells, and glandular epithelial cells, play an important role at the maternal-fetal interface and produce chemokine gradients to recruit decidual leukocytes. Decidual stromal cells were differentiated from endometrium fibroblast-like precursor cells by estrogen and progesterone. They also have immunological competence.

Following implantation, the trophoblast invades the uterine lining and ultimately erodes the spiral arteries (77, 78). Thus the trophoblast forms contact with the maternal immune system during the early pregnancy period. Soon afterward, endometrium decidualization and placentation ensue. The trophoblast epithelial cells derived from the trophectoderm of the blastocyst are therefore regarded as the functional unit of the placental villus. Without them, the placenta cannot be established as a pivotal immune-crosstalk barrier and the site for the sole exchange of gases, nutrients, and waste between maternal and fetal tissues.

There are various types of trophoblast continuously developed when the blastocyst has anchored maternal decidua. The innermost progenitor trophoblasts, termed villous cytotrophoblasts (VCT), are mitotic and are continuously proliferative. VCTs fuse to constantly replenish the two layers of multinucleated syncytiotrophoblast (SCT). The SCT has a highly polarized epithelial layer densely covered with microvilli, which increases its surface area five-to-seven fold (78). Secretion of important molecules is the predominant function of the SCT. The SCT forms an excretive barrier against toxic substances circulating in the mother and also participates in nutrient and oxygen transport and secretion of hormones and proteins, which are released into maternal circulation and then drive the metabolic adaptations. The SCT also expresses the neonatal Fc receptor (FcRn) involved in maternal-fetus immunological communication (79). In addition, while HLA class I mRNA is found in the SCT, it does not express HLA molecules, and so acts as a protective immunological barrier. Moreover, SCT can also secrete apoptosis-associated molecules like Fas ligand (FasL) and TNF-related apoptosis-inducing ligand (TRAIL) (80). Cytotrophoblasts cell columns (CCCs) occur when anchoring villi encounter the decidua. A CCC is deemed to form an epithelial-mesenchymal transition (EMT) layer and is the interim switch from VCT to EVT. Terminal mono-nucleated EVTs are differentiated in the outer layer of CCC and bathed in maternal blood. The EVT populations were roughly divided into three types: interstitial, intramural, and endovascular EVTs. Endovascular EVTs are the most important (81). They invaded around and replaced the uterine spiral arteries and finally remodel to a new vascular shape of high conductance at low pressure in favor of the ongoing development of the fetus irrigated by abundant maternal blood flow. EVTs are also the essential trophoblasts for the fetus to tolerate the mother due to the existence of HLA-C, HLA-G, HLA-E, and HLA-F. Certainly, these HLA molecules expressions were regulated by the pregnancy microenvironment like hormones, cytokines, growth factor, signaling molecules, and nuclear receptors (82). Other placental cells are also important for the construction of the maternal-fetal immune environment. Hofbauer cells, one of the immune members, can prevent the fetus from vertical infection and nutrient interchange.

Actually, the cellular constitution is fairly complicated, beyond what has been discussed above. Myeloid decidual immune cells, stromal cells, and glandular epithelial cells, which are derived from the mother, predominantly interact with fetal EVTs and thereby generate multifarious cytokines, growth factors, and hormones. All those together comprise the unique maternal-fetal immunological microenvironment to support successful pregnancies.

#### 2.2.2 The Mother-Baby Immune Trafficking: Fetal Immature Antigens and Maternal Immune Inertness?

The physiological structure of the maternal-fetus interface is fundamental to mother-fetal immunologic cross-talk. In regard to immune communication from ‘Medawar’s Paradox’, fetal immature antigens play a part, and another participant is maternal immune inertness. However, studies now suggest that this is not the case, since it appears that fetal antigens are not entirely immature and the maternal immune system can generate immune responses and respond robustly to various pathogens.

Certainly, fetal immature antigens are a significant factor and are not recognized or attacked by maternal immune circulation. This poses the question as to why fetal-derived antigens do not initiate the immune response. Firstly, paternal-derived alloantigen recognition is impeded on account of trophoblastic non-expression of part-classical HLA class I molecules. HLA-A and –B are crucial to match in transplantation against immune rejection. Without HLA-A and -B, T cells and NK cells cannot perform cytotoxic functions. It would be expected that maternal APCs ought to arrange and present antigens to activate T cells and NK cells. However, this indirect antigen recognition does not happen. One reason might be the slight contribution of dMφ and DC populations residing in decidua. Secondly, trophoblasts and especially EVTs express a unique set of HLA antigens. The presence of HLA-C and -G prevents dNK cells from attacking trophoblasts. This is supported by *in vitro* studies, where HLA-C acts as a protective molecule and prevents dNK killing (34). The interaction of HLA-C with KIR2DS1 expressed on the surface of dNK cells further actives the production of salutary cytokines (83). HLA-G is also the main protective molecule against the cytotoxic effect of T cells by connection with KIR2DL4, ILT2, and ILT4. sHLA-G is secreted to downregulate CD4+ T cell proliferation, as well as to induce the apoptosis of activated CD8+ T cells. Membrane-bound HLA-G (mHLA-G) combine with inhibitory receptors presented in dNK cells and APCs and subsequent make those immune cells result in anergy, and eventually restrict the attacking of mother against fetus. Moreover, in *in vivo* or *in vitro* studies, HLA class II antigens were not expressed in any early trophoblast subpopulations and but, interestingly, in in uterine macrophages after mid-trimester (84). Despite this, the contribution of fetal antigens to maternal-fetal immunological tolerance are not well understood. The evidence suggests that the fetal immune system response to the mother was established until mid-trimester and term-trimester. Fetal immature antigens are still not overlooked.

Although the maternal immune circulation is not subjected to immunological inertness, immune cell populations are elaborately altered, both locally and systemically. Decidual lymphocyte populations have shown locally different phenotypes: γδ+T cells and CD4-/CD8- T cells have been described in pregnant uteruses (85); dNK cell was CD56+CD16- type as previously described (86); monocyte-macrophage populations have a bias towards Mφ2, that produces an anti-inflammatory effect (87).

Tregs and Th cells are the vital mediators of maternal adaptive immune reactions. The CD4+CD25+FoxP3+Tregs population increases just before the ovulatory period, a stage where the extraneous paternal-derived antigens might be exposed to the maternal immune system (76). These Tregs continue expanding by the stimulation of estrogens, progesterone, and trophoblastic cytokines until implantation occurs. When HLA-G+ EVT and CD4+ T cells are co-culture, the number of cells expressing a Treg phenotype was increased (88). These Tregs were lasting after delivery and were detected in subsequent pregnancies. In a mice model, selectively killing these Tregs could result in a low birthrate of offspring. Apart from the secretion of inhibitory cytokines, Tregs play a part in immune regulation through contact with targeted cells with T cell receptors (TCR) and so on. Costimulatory molecules such as PD-1/PD-L1, TIM-3, CTLA-4/CD86, and CTLA4/CD80 invoke a common negative capability of antigen presentation by Tregs (89–91). PD-L1 upregulation was detected in SCT (92). Its downregulation results in Tregs apoptosis and Th1 increase. This suggests that the PD1/PDL1 checkpoint contributes to the maintenance of maternal-fetal immune-tolerance. Cytotoxic T lymphocyte (CTL) is another adaptive immune mediator. Fetal-specific CD8+ T cells increase during pregnancy and the post-natal period (93). These effective CD8+ T cells could induce target cells to undergo apoptosis or lysis *via* the Fas/FasL pathway, as well as perforin and granzyme attacks. Further, downregulation of the CD8+ T cell population has been linked to pathological pregnancy conditions, particularly preeclampsia (94). Using a transgenic mice model, research has shown that T cells that recognized paternal HLA class I antigens were selectively depressed. During pregnancy, there is a shift in the balance of the Th1/Th2 profile towards more dominant Th2 cytokine-producing lymphocytes, which was the initial theory for explaining the systemic changes in maternal immunity. However, more recent studies have revealed that more complicated systemic changes occur than just a simple shift towards a Th2 profile. For example, IL-2 is essential for the proliferation and differentiation of T cells. IL-2 is mainly produced by Th1 cells, Tregs, and CD8+CTL, but not Th2 cells, and is increased during pregnancy (95). Th17, a subpopulation of CD4+ T cell producing IL-17, has also been reported in pregnant uteruses (96). It is a pro-inflammatory cell and plays a role in eliminating pathogenic microorganisms. The Th17/Treg ratio is associated with pregnancy outcomes such as recurrent implantation failure (96). However, recent research in recurrent implantation failure has shown that the Th17/Treg ratio was susceptible to Treg enhancement and Th17 diminishment using hydroxychloroquine treatment but the pregnancy outcomes were not significantly improved (97). In addition, Breg, the subpopulation of the CD19+CD24hiCD27+ phenotype, were expanded by producing IL-10. They act as a suppressor of undesired immune responses from maternal T cells.

dNK cells and dMφs are two dominating immune-regulatory cells that play a key role in the innate immune system. As mentioned, these two types of cells occupy the majority of immune cells in the decidua. dNK cells act as an essential immune regulator for trophoblast invasion and spiral artery remodeling. The expressed receptors interact with the HLA ligands presented in EVTs and thus form kinds of combinations, such as ‘KIRs/HLA-C’, ‘CD94/NKG2A/HLA-E’, or ‘KIR2DL4/HLA-G’. Through these suppressive combinations, dNKs cannot activate cytotoxic mechanisms and facilitate trophoblastic lysis at the maternal-fetus interface. On the contrary, these re-educated dNK cells have low cytotoxicity and promote growth through the secretion of pro-angiogenic factors. These behaviors differ from maternal peripheral pNK cells, with efficient targeted-cell killing and cytotoxic activity. HLA-G presented on EVTs can bind to KIR2DL4, ILT2, or ILT4 expressed on dNK cells surfaces, leading to the secretion of IL-2, IL-8, TNF-α, GM-CSF, macrophage inflammatory protein (MIP), vascular endothelial cell-C (VEGF-C), and placental growth factor which are beneficial for pregnancy. Interestingly, high expression of NKG2C and LILRB1 has been observed in some dNK cells after repeated pregnancies. The author has subsequently named this subpopulation of dNK cells Pregnancy Trained decidual NK cells (PTdNKs). PTdNKs have open chromatin around the enhancers of IFNG and VEGFA and thus enhance performance for vascular remodeling and angiogenesis in subsequent pregnancies (73). Hence, the ‘memory’ of dNK cell education and license should be further investigated. dMφs also act as primary APC and have anti-inflammatory and angiogenesis phenotypes at the maternal-fetus interface. Like Tregs, they can also produce IDO to inhibit T cell activation. dMφs exhibit phenotypic elasticity and display Mφ1 in preimplantation but shift to Mφ2 after placentation (98). Mφ1 cells can aid in positive immune responses by presenting antigens and secreting pro-inflammatory cytokines or chemokines. They are intimately involved in immune defense and surveillance. In contrast, Mφ2 cells have a weakened ability to present antigens and produce inhibitory factors such as IL-10 and TGFβ. Mφ2-driven upregulation of CD85j on NK cells can induce the generation of hypo-responsive NK cells. Mφ2 cells can also present higher amounts of HLA-G than Mφ1 cells and thus limit NK cell effector ability (99). Nevertheless, how Mφ2 cells precisely enhance the chances of successful pregnancy requires further study.

The roles of dDCs and mast cells are less clear. However, their presence may contribute to successful placentation. The recruitment of DCs and the persistence of dMφs are associated with preeclampsia. Uterine mast cells are higher during pregnancy than non-pregnancy.

In addition to immune cells, other factors exist in the microenvironment of the maternal-fetal interface that influences tolerogenesis.

Hormones like progesterone and Prostaglandin E2 are increased during pregnancy and are known to participate in the maternal immune response. Human chorionic gonadotropin (hCG), a gestational-specific hormone, is has been shown to stimulate the production of regulator B (Breg) cells. Subsequently, Bregs produce IL-10, IL-35, and TGF-β and are powerful immunosuppressive regulators. Progesterone has been shown to inhibit Toll-like receptor-induced cytokine production, as well as promote Th2 immune responses (100). It can also induce anti-inflammatory factors. These conditions are advantageous for promoting and sustaining pregnancy. Prostaglandin E2 is produced by Mφs and decidual cells. It contributes to ameliorate the poor proliferation of lymphocyte.

FcRn is a major histocompatibility complex (MHC) Class I-related receptor that interacts with antibodies of the IgG class *via* the constant or fragment crystallizable (Fc) region (101, 102). It is the placental transporter of IgG from mother to fetus. FcRn acts not only in the transfer of protective immunity but also tolerogenic molecules as well. For instance, in a transgenic mice model, FcRn-dependent trans-placental transport of Fc-hemagglutinin induced tolerance *via* antigen-specific regulatory T (Treg) cells (103). Fc fused proinsulin was injected into pregnant mice to protect fetuses from developing postnatal autoimmune diabetes. These FcRn fused proteins resulted in the emergence of antigen-specific thymus-derived CD4^+^ T_reg_ cells and impaired cytotoxic CD8^+^ T cells (104). Also, in another study focused on tolerance to food allergy, FcRn-mediated IgG immune complex transfers to the newborn induced antigen-specific Foxp3^+^ T_reg_ cells via the presence of TGF-β (105).

Fetal and maternal micro-chimeras are found in the tissues and circulation of mother and baby, respectively. Long-term micro-chimeras are significant for maternal immunological tolerance. Maternal micro-chimeras have been considered as a mechanism to induce maternal immune tolerance towards fetal inherited paternal antigens (IPAs). They have a bidirectional role, being linked to the development of autoimmune disorders in women and the protection against some cancers in parous women (106). Conversely, fetal micro-chimeras can induce fetal tolerance towards non-inherited maternal antigens (NIMAs). Accumulating evidence suggests that IPAs and NIMAs define the degree of HLA mismatch between mother and fetus (107). However, mothers bear IPAs for a short-lived period, while babies carry NIMAs for their whole lives. Incidentally, the relationships between micro-chimeric IPAs/NIMAs and tolerance have been widely studied in hematopoietic stem cell (HSC) transplantation. The low morbidity of graft-versus-host disease is associated with HLA-haploidentical HSC transplantation from a micro-chimeric IPAs/NIMAs-mismatched donor. The mechanism behind this phenomenon might be related to the deletion of IPA/NIMA reactive T cells and upregulation of Tregs (108). Whether there are other contributing factors influencing tolerance, such as cell-free fetal DNA and telomere mimicry, remains the subject of ongoing research (109).

The low tryptophan level and high progesterone standard seem to be beneficial for fetal receptivity. Tryptophan might be disintegrated by IDO, which is released by dMφs, monocyte-derived DCs, SCTs, and EVTs. Evidence suggests that IDO is important for downregulating maternal T cell responses (110). Additional tryptophan catabolism modulated by kynurenine, 3-hydroxykynurenine, and 3-hydroxyanthranilic acid also affects T cells apoptosis. Members of the B7 family, specifically expressed in the SCT, can interfere with the activation of maternal circulating lymphocytes. The TNF superfamily, such as TNF-α, FasL, and TRAIL, induce apoptosis of potentially cytotoxic T cells or support the production of maternal or fetal antibodies, and thus have a beneficial effect on pregnancy. Complement proteins, such as membrane cofactor protein, decay-accelerating factor, and membrane inhibitor of reactive lysis also make a protective impact on pregnancy by initiating regulation. Chemokine C-C motif ligand (CCL) and its receptors: C-C chemokine receptor (CCR) and C-X-C chemokine receptor (CXCR) is significant for maternal-fetus immunological cross-talk and placentation by recruiting decidual immune cells and domesticating their function.

The maternal systemic immune response has dual markers of both activation and inhibition. However, selective inhibition or modulation of maternal immune responses may occur instead of generalized immunosuppression.

Consequently, current studies suggest that mother-baby immune trafficking is intricate. Even without a macroscopic exchange of blood between mother and fetus, the placenta allows the bidirectional traffic of cells, mainly trophoblastic stem cells and leukocytes (107).

### 2.3 The Perplexing Transplant Immunological Tolerance and Pregnant

Similar but different to pregnancy, the process of transplantation also expresses alloantigens. The usual obstacle, which known is as immunological rejection, hinders HSC and organs transplantation. Therefore, immune suppression is applied to clinical therapeutics. Induction of immunological tolerance is also needed for keeping the graft alive. Although tolerance in transplantation and pregnancy is managed differently, researchers can learn from each other.

How is transplant tolerance induced? Firstly, we need to understand how transplant rejection occurs. The transplanted organ is directly attacked and damaged by alloreactive CD8+ T cells through cytotoxic mechanisms (111). Subsequently, these activated T cells secrete cytokines and produce alloantibodies. Following these events, the classical complement pathway is initiated to cause chronic damage and failure of the transplanted organ. Meanwhile, immune cells express pathogen-associated pattern recognition receptors (PRR) to recognize structural units on pathogens and damage-associated molecular patterns (DAMPs). PRRs encounter DAMPs and lead to an inflammatory response involving the production of ILs, TNF, and chemokines. These factors alter the permeability of vascular endothelial cells and lead to the recruitment of antigens, APCs, and other leukocytes into the graft. Thus, the immune rejection has been fully triggered (112). The relationship between HLA typing crossmatch and transplant outcome is generally accepted in immunology. Thus, evaluation of the HLA molecules involved in the transplant is useful. HLA-A, -Bs and –DR represent the regular HLA typing crossmatch genes to test before transplantation.

**Figure 3:**
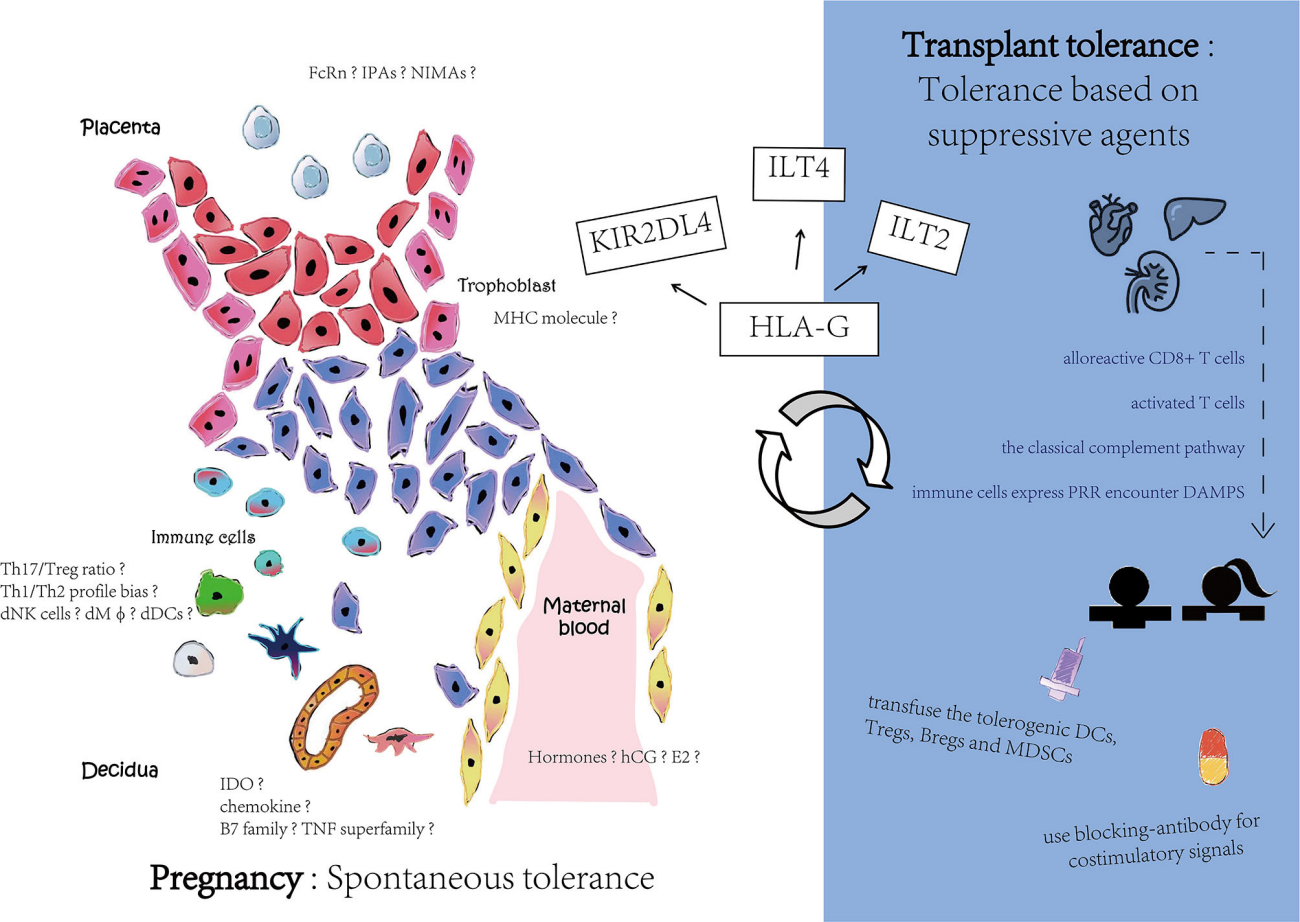
Different mechanisms in pregnancy and transplant tolerance. Pregnancy has a spontaneous tolerance while transplant tolerance is based on suppressive agents. Although there are still many speculated tolerance mechanisms among pregnancy, HLA-G interaction with its receptors may contribute to maternal-fetal interface and reward to organ transplant.

Compared with universal immunological suppression by medicines, the application of persistent and stabilized immune tolerance requires an urgent solution. For inducing central tolerance, intrathymic injection or allothymic transplantation has been used. Allogeneic micro-chimera of HSC is established by high-dose systemic radiation and unceasing immunosuppressive agent treatment. For inducing peripheral tolerance, there is an effective strategy for prolonging graft survival in which blocking costimulatory signals induces alloreactive T cell inactivation. For instance, using anti-CD40L monoclonal antibodies, the CD40L-CD40 costimulatory pathway impeded the mediation of activated T cells and B cells. Another strategy is to transfuse the tolerogenic DCs, Tregs, and MDSC to induce alloreactive T cell apoptosis and anergy. However, all of these strategies for inducing transplant immunological tolerance need external immunosuppression, that is, they induce tolerance firstly based on immunosuppress agents, while in pregnancy the tolerance is spontaneous (see **Figure 3**). Thus, transplant tolerance is a pathological condition while pregnancy tolerance is a physiological status.

HLA-G is thought to be an IC that links to ILTs to form a signaling pathway (31). Recently, it has also been extensively studied in transplantation (113, 114). ILTs might recognize the distinguished different structures of HLA-G: ILT2 is prone to recognize the β2m-associated structure, while ILT4 interacts with non-β2m-associated structures. Specifically, in heart transplants, 18% of patients have an HLA-G presence (114). The HLA-G- positive patients have a comparatively lower incidence of acute rejection than the HLA-G-negative group. Other research studies also support the notion that decreasing levels of HLA-G might be linked to higher transplant rejection rates (115). Higher HLA-G expression also correlates with a higher population of CD3+CD4-Foxp3- Treg cells, which might result in lower rates of transplant rejection (116). HLA-G5, one of the soluble HLA-G isoforms, can suppress the proliferation of T cells during the alloreactive CD4+ T cells-derived allogeneic response. HLA-G5 is also associated with the occurrence of graft versus host diseases (117). In addition, sHLA-G can regulate B cell functions to prevent a humoral rejection in a lung transplant recipient cohort (118).

Immunological tolerance seems to decrease the risks of cancer, infection, and cardiovascular disease after transplantation (119). There is a growing interest in utilizing HLA-G as a therapeutic modality in clinical transplantation (113) which will comprise the context of later discussions.

## 3 Involvement of HLA-G in Maternal-Fetal Immune-Tolerance

Since the induction and maintenance of robust immunological tolerance has been the holy grail of immunology, it is significant for the development and application of IC in transplantation, tumor treatment, and autoimmune diseases (120). IC inhibitor therapy is safe and well-tolerated regardless of age, such as anti-PD-1 and anti-CTL4 antibody administration (121). The age has arrived to think more about how to exploit tolerance in immunotherapy! HLA-G, as the novel IC, is firstly activated at the maternal-fetal interface. It is uniquely presented in EVTs and interacts with other cells and, perhaps, other HLA molecules in the maternal-fetal microenvironment. Research had revealed that sHLA-G levels were decreased in term pregnancy, while HLA-C levels were increased until labor. HLA-E was only found in early pregnancy. HLA-F remains weakly expressed until term (122). The understanding of HLA-G roles in the mechanisms of maternal-fetal immune tolerance is extremely important.

### 3.1 HLA-G Acting in the Pregnancy Microenvironment

For the sake of facilitating successful pregnancy, HLA-G molecules can interact to inhibit or activate receptors expressed by maternal immune cells (123–127) (see **Table 2**). Studies of HLA-G isoforms have principally focused on HLA-G1 and HLA-G5. Accordingly, the investigations of maternal immune cells have focused on T cells, dNK cells, and Mφs.

ITL2 receptors are mainly present in T cells, B cells, Mφs, monocytes, and some NK cells. They seem to be the most prominent inhibitory receptors that interact with HLA-G. Meanwhile, ILT4 receptors are expressed on NK cells, Mφs, and DCs. T cells and NK cells, two major immune cells at the maternal-fetal interface, receive an inhibited killing signal when ILT2 or ILT4 bind to HLA-G. ILT2 can only bind to β2m-associated HLA-G1 and -G5 isoforms, but ILT4 preferentially recognizes β2m-free HLA-G2 and -G6 and also β2m-associated HLA-G. HLA-G also inhibits CD8+ T cells’ killing capacity by downregulating the granzyme B expression. CD8 is expressed by both cytotoxic T cells and some NK cell subsets. In studies of women with cytomegalovirus infection, HLA-G targets the CD8αβ heterodimer with TCR and generates an allogeneic CTL response. However, CTL cell responses are not elicited by trophoblasts, which is attributed to the absence of polymorphic HLA-A and HLA-B. The peptides derived from cytomegalovirus can stabilize surface HLA-G expression and active T cell responses in transgenic mice. Both membrane and soluble HLA-G regulate CD8+ T cells by eliminating alloreactive T cells, triggering Fas ligands to induce CD8+ cell apoptosis and Fas/FasL pathway-related T cell death. To some extent, the prevention of the cytotoxic activity of CD8+ T cells against target cells through HLA-G is independent of the induction T cell apoptosis (128). HLA-G1 and its soluble counterpart HLA-G5 protect potential target cells from lysis by antigen-specific cytotoxic T cells (129). HLA-G1 also prevents CD4+ T cells expressing an immunosuppressive phenotype from proliferation. In an *in vitro* study, CD4+ and CD8+ T cells could acquire a similar HLA-G1+ phenotype through trogocytosis. CD25+ T cells also acquire HLA-G1+ phenotype in the same manner but differ functionally. These types of CD25+ T cells do not secrete sHLA-G5 and IL-10. They show the immune suppressive function in a cell contact dependent manner. HLA-G+ Tregs regulate immune responses by accumulating at inflammatory sites. CD4+HLA-G+ Tregs are observed to increase in peripheral blood in pregnancy compared to non-pregnant controls.

Additionally, Mφs also obtains a tolerogenic phenotype through expressing ILT2 or ILT4 receptors interact with HLA-G ligands. Then, they reduce the expression of CD80 and CD86 and increase IDO levels. dMφ cells are activated by the expression of CD11c and CD86 (130, 131). They secrete anti-inflammatory cytokines such as IL-10 and other markers related to immune evasion such as TGF-β, B7-H1, ILT3, DC-SIGN, MS-1, and factor 13. DCs, another APC, can also be affected by HLA-G. HLA-G induces DCs to differentiate into tolerogenic DC-10 cells (132, 133). HLA-G tetramers only act on DCs and this combination was found to strikingly diminish the graft rejection. Passingly, CD14+ Mφs and CD83+ mature DCs in decidua produce the lymphocyte inhibitory molecules for reducing T cell responses (132). However, the relationship between HLA-G and APC seems to depend on the inflammatory condition. This requires more investigations into their relation in non-inflammatory conditions.

KIR2DL4 is primarily expressed in dNK cells (45). It is a controversial receptor with uncertain inhibition or activation functionality, and binds to HLA-G monomers or dimers. These ‘HLA-G/KIR2DL4’ combinations are able to result in the production of pro-inflammatory cytokines like IFN-γ for driving cells into an immunosuppressive phase (134). KIR2DL4 could be also observed in mast cells recently (135). It was involved in Janus kinase-signal transducer and activator of transcription (JAK/STAT) signaling pathway association with trophoblast invasion and angiopoiesis by HTR-8/SVneo cell line.

The combination of the HLA-G and CD94/NKG2A heterodimer presented on the surface of NK cells prevents NK cell cytolysis in transfection-based assays (136). However, this observation has not been substantiated *in vivo*. The true ligand for CD94/NKG2A is actually HLA-E, which utilizes a leader peptide derived from HLA-G and permits HLA-G expression. Repressed HLA-G expression by overexpression of miR-152 has been shown to lead to increased NK cell-mediated cytolysis of JEG-3 cells, and also affects the invasion of JEG-3 cells (16, 137).

In general, cells expressing HLA-G and its receptors are found in co-localization, but the mechanisms responsible for this adjacency are still unknown and need further investigation. Anyway, HLA-G definitely contributes to the acknowledged mechanisms of maternal-fetal immunological tolerance throughout pregnancy. Other HLA molecules are probably mutually linked to each other.

### 3.2 HLA-G and Pathological Reproduction

Maternal-fetal immune-tolerance has clinical significance for pathological conditions of pregnancy. To date, immunotherapies are only effective in a fraction of women with recurrent implantation failure or recurrent spontaneous abortion. This remains one of the most controversial issues in ART. Different immunomodulation strategies have been used without a sound scientific basis or a sound clinical trial design (138). In addition, most clinical studies have ignored patient heterogeneity. Hence, it is urgent to revisit and reinvestigate the breakdown in fetal-maternal tolerance in pregnant women, particularly those with obvious pathological conditions such as recurrent miscarriage, fertilization failure, and preeclampsia.

Several studies have reported a higher chance of successful IVF treatment for women with higher blood levels of sHLA-G. In contrast, there is a lower success rate in women where the fetus expresses an HLA-G allele with a 14bp insertion in 3’UTR (139–141). The insertion leads to low expression of HLA-G, and a higher risk of recurrent spontaneous abortions (142). For a successful outcome, HLA-G expression in the preimplantation endometrium appears to be more important for women requiring assisted conception than a matched comparative fertile group (143). A higher expression level of sHLA-G in endometrium is associated with an increasing number of CD56+ NK cells and the outcome of the following IVF cycle. Further, after IVF treatment, high sHLA-G levels detected in the embryo culture medium correlate with a higher success rate of implantation and pregnancy (144).

Studies have also shown that HLA-G might be regulating vascularization. Since preeclampsia is an angiogenesis defect disease and is well known for the ‘two-stage’ hypothesis under oxidative stress, HLA-G also has an effect on preeclampsia with low expression and 14bp ins/del polymorphism (145).

An HLA-G null allele called G*0105N is caused by a single base pair deletion at +1597, and is associated with an increased risk for recurrent miscarriage. However, this allele does not encode functional HLA-G1 or HLA-G5 isoforms and also can be found in healthy individuals. HLA-G2 and -G6 seem to be substituted in these cases due to their similar location to HLA-G1 in many cells. HLA-G*0113N, another null allele, is also reported to relate to unexplained recurrent abortion. It is caused by a single exchange of cytosine to thymidine at position 54. It is interesting that the frequency of HLA-G*0113N is described only in African people whereas HLA-G**0105N is often in African and Indian people.

## 4 Perspective

HLA-G polymorphisms were first reported to be associated with pregnancy complications in the early 21^st^ century (146). Hereafter, there has been a stream of research publications focused on understanding the relationship between HLA-G and human pathological pregnancy. For instance, the soluble HLA-G phenotype may be a useful diagnostic with clinical therapeutic utility for women with infertility or pregnancy complications. However, due to the lack of applicable in-vitro models, investigations to ascertain the role of HLA-G are restricted and remain controversial.

Primary EVTs, which specifically express HLA-G under normal physiological conditions, can be isolated from the first trimester placental villus. However, they are vulnerable and poorly adhere to plastic after 24h. When culturing *in vitro*, they gradually cease to proliferate and lose their natural phenotype in 3-4 days. Indeed, they are overgrown by mesenchymal cell contaminants (78). Thus, they are unsuitable as an *in vitro* model. In regard to animal models, the orthologous HLA-G gene is absent in mice, although Qa-2 is considered the putative functional homolog of human HLA-G (147). The only HLA-G transgenic mice produced to date express H-2kb/HLA-G, however, HLA-G is widely expressed in almost all somatic cells and thus is not particularly a useful model (148, 149). Moreover, HTR-8, the immortalized first-trimester trophoblastic cell line, is also unable to express the HLA-G protein. Likewise, other trophoblastic cell lines, such BeWo, JAR, and SW.721 also do not express HLA-G. However, the JEG- trophoblastic cell line, derived from choriocarcinoma rather than normal placental villus, does expresses the HLA-G protein.

Recently, the establishment of trophoblast organoids from human placental tissue is an alternative *in vitro* model for studying the biological function of HLA-G (78). Accordingly, a human endometrium organoid was formed after long-term culture in a hormone-responsive environment (150, 151). With the availability of similar models, it will be now possible to assess the precise mechanisms by which HLA-G contributes to maternal-fetal immune-tolerance. The maternal-fetal interface is analogous to the immunologically privileged sites during pregnancy. Reconstituting this immunologically microenvironment is meaningful. One of the available ways is combine the trophoblast organoid and endometrium organoid in microfluidic device within immune cells resided in uterus.

The induction of micro-chimeras may be a useful strategy for curing diseases related to a failure of immunological tolerance. Several clinical trials have shown the effectiveness of various hematopoietic micro-chimeras for the development of transplant tolerance (152, 153). Transient, mixed-donor, and full-donor micro-chimeras were induced when infusing donor hematopoietic stem cells. Micro-chimeras are found as a result of maternal-fetal cellular trafficking during pregnancy. More attention should be given to understanding the role of fetal micro-chimeras in pregnancy. Fetal micro-chimeras are highly associated with maternal autoimmune disease and are found in patients with lower morbidity rates for cancer (154, 155). In a study of women with scleroderma, compared to disease-free women, there was reduced sHLA-G expression and higher quantities of persistent fetal micro-chimeras in the circulation (156). Interestingly, high levels of fetal micro-chimeras do not lead to higher levels of sHLA-G in offspring (157). The relationship between HLA-G and fetal micro-chimeras requires further studies. Induction of EVT-derived micro-chimeras containing the HLA-G paternal haplotype (that is, partly analogous fetal micro-chimeras) might contribute to the therapy for immunopathologic pregnancy such as recurrent miscarriage.

Enhanced immunological tolerance based on stem cell transplantation is another interesting area that warrants further research. Our team established a human embryonic stem cell (hESC) model that expresses HLA-G1 and was capable of normal differentiation into the three germ layers (158). Neural progenitor cells, one of the hESCs-differentiated cell types, retained higher levels of HLA-G1 and could enhance the immune tolerance ability by suppressing T cells proliferation and NK cells cytotoxicity (159). Further, the neurons derived from HLA-G1-H1 hESCs could also attenuate the release of the pro-inflammatory cytokines IL-1β and IFN-γ from lipopolysaccharide-stimulated BV2 microglia (160). In another earlier study, we overexpressed HLA-G1 in hESCs and observed their ability to inhibit mixed T-lymphocyte proliferation. However, the usage of hESCs and pluripotent stem cells (iPSCs) is hindered by ethical concerns. Nowadays, mesenchymal cells (MSCs) transplantation is a new promising strategy for tolerance induction (161). MSCs are convenient to isolate, have strong self-renewal abilities, and have multi-potent differentiation abilities (162). Several studies have reported the ability of MSCs to regulate mHLA-G and sHLA-G antigens when MSCs were co-cultured with activated peripheral blood mononuclear cells or directly activated by exogenous IL-10 (163–165). Moreover, when co-transplanted with HSCs, MSCs produce sHLA-G to prevent graft versus host diseases (GVHD) (166). In another study, increased HLA-G expression by human amniotic membrane-derived mesenchymal cells was linked to increased cardio-myogenic trans-differentiation efficiency (167). MSCs selected for specific HLA-G gene status have also been successfully used for treating GVHD (168).

Even so, we should not ignore the traditional therapeutic modality related to immunological tolerance about HLA-G. Treg cell therapy remains a promising strategy for the establishment of tolerance. The therapeutic applications of dNK cells are under investigation. Currently, more and more new technologies are being developed, such as high dimensional flow cytometry, single-cell RNA sequencing, knockout models or antibody-based depletion, nanoparticles, and exosomes (113, 169, 170). How to utilize these new technologies to study the biological function of HLA-G, as well as the clinical therapy through HLA-G-induced immunological tolerance, ought to be further explored. Using single-cell RNA sequencing to define the transcriptomic landscape of placental trophoblasts derived from cultured human blastocysts, the migratory trophoblast transcriptome at D12 indicates the IFN-related markers are associated with the up-regulation of HLA-C, -E, and –G (171). However, the research lacks maternal decidual cells and fibronectin, and thus might be somewhat different from the events of normal pregnancy *in vivo*. Organoids connected single-cell RNA sequencing could give a complete indication of what goes on *in vivo*.

## 5 Conclusion

Our understanding of maternal-fetal tolerance has been aided by understanding the mechanisms of peripheral tolerance and how the immune system responds to a transplant or developing tumor. HLA-G is an essential regulatory molecule that interacts with specific immune cells to induce a state of fetomaternal immunologic tolerance to protect the pregnancy. Research should now be focused on developing safe strategies to selectively suppress or enhance immune responses regulated by HLA-G that can be applied to women with pathological pregnancy disorders, as well as patients receiving transplants or with a life-threatening tumor.

## Author Contributions

All authors listed have made a substantial, direct, and intellectual contribution to the work and approved it for publication.

## Funding

This review research was supported by National Key Research and Development Program of China (No. 2018YFC1003100).

## Conflict of Interest

The authors declare that the research was conducted in the absence of any commercial or financial relationships that could be construed as a potential conflict of interest.

## Publisher’s Note

All claims expressed in this article are solely those of the authors and do not necessarily represent those of their affiliated organizations, or those of the publisher, the editors and the reviewers. Any product that may be evaluated in this article, or claim that may be made by its manufacturer, is not guaranteed or endorsed by the publisher.
